# Louisiana National Life Assurance Society, New Orleans

**Published:** 1911-04

**Authors:** 


### TEXAS MEDICAL JOURNAL.

Established July, 1885

F. E. DANIEL, M. D., - - - - - - Editor, Publisher and Proprietor

Published Monthly.—Subscription $1.00 a Year.

VOL. XXVI AUSTIN, APRIL, 1911. No. 10

The publisher is not responsible for the views of the contributors.

### Original Articles.

### Louisiana National Life Assurance Society, New Orleans.

A meeting of the Medical Section of the American Life Convention was held in New Orleans, February 23-25, upon which occasion our esteemed correspondent, L. Sexton, B. S., M. D. (New Orleans), read a valuable paper, from which we make the following extracts:

Dr. Sexton said:

"If starvation has killed its thousands, over-eating and drinking has killed its ten thousands. Overweight carries the suggestion of over-feeding or over-drinking. The experience of life insurance companies in dealing with such risks has been hazardous in the extreme. There is a French adage, that 'We dig our graves with our teeth.' While over-eating is not so damaging as intemperate drinking, it is, nevertheless, one of the common cause of short-life history. This can be readily understood when we contemplate the fact that the average well-to-do person eats twice as much as his necessities require. The food is often very improperly cooked and masticated, in addition to the over-eating. Gladstone, who lived to be 97 years old, was reputed to have masticated fifteen times every morsel which he swallowed, so that the food was thoroughly mixed with the salivary juices, and in an absorbable state before it entered the stomach at all. Over-eaters, drinkers and gormandizers should remember that the twenty-nine feet of alimentary canal is the sewer of the system, and that it is more risky to neglect the one within you than the sewer on your premises. The large meat eaters and wine drinkers should know that food undergoes the same decomposition within this sewer as it would undergo if improperly kept outside of the body. The ptomain and other poisonings of which we read so often in the daily press are but records of this decomposing food within the body. These, however, are the acute results which impress themselves forcibly upon the mind, but it is the slower results upon the liver, the hardened arteries and over-choked kidneys, dilated stomach, and gaseous distensions, that are some of the secondary results of this over-feeding, which are only too often referred to other causes. As heart and kidney diseases very often go hand in hand with intemperance in both eating and drinking, it is the overloading of the system with indigestible food and drink that is very often the starting point of many of the sudden deaths which life insurance companies are called upon to pay. Gout and rheumatism are two other diseases high in the mortality tables that are very often traceable to the over-eating and drinking of the subject under consideration. The average man fifty years weighing 200 pounds and less than 6 feet tall is almost sure to have hyaline or granular casts in his urine, if he has been an excessive eater or drinker. The lack of elimination in such subjects on account of constipated habits and improper action or lack of action of the kidneys or liver, have led to numerous fatalities that otherwise might have been averted. It is the opinion of some medical directors that no man with an abdominal measurement of over forty-seven is an insurable risk. Yet the medical directors are constantly asked to pass upon risks favorably that even an army recruiting station flatly turn down. The army and navy rules are only two pounds to the inch up to five feet, seven inches; after this height, seven pounds may be added to each inch, and still be within the range of acceptance. If you will exclude the lightweights with consumptive histories or taints in the family, they have really proven more desirable risks than have these heavyweights, who are addicted to intemperance both in eating and drinking.

"Malaria, like many other diseases, has had much of the venom or sting extracted from it. You remember the fearful mortality that almost decimated the laborers under De Lesseps in his efforts to excavate the Panama Canal. The mortality at that time from both malarial and yellow fevers reached unprecedented records. These diseases as much as political graft and inefficiency caused the abandonment of the project by the French government after twenty years' of disastrous experience.

"As a matter of course, you all know the history of Dr. Gorgas' sanitary work in Panama, which has reduced the mortality on the isthmus to 15 per 1000, or about equal to the mortality rate in our best sanitated cities in the United States. What has been done in Panama has, to a limited extent, been duplicated in our Southern States. Not so thoroughly, as a matter of course, because one is done under military regime and the other by sanitary inspectors and boards of health, with less power of enforcement of regulations. Another condition which has helped on the isthmus has been the issuing of rations of quinine just the same as food. This compulsory prophylaxis or taking quinine by the ton has helped to eradicate the malarial plasmodium. The drainage of the swamps and lakes to destroy the breeding places of mosquitoes has also had much to do with its reduction, but so long as a number of people were acting as hosts as between the mosquito and the next man to be inoculated, there could be no definite stamping out until the population of the zone had been practically cinchonized.

"In our Southern States the great majority of the anophiles mosquitoes perish or hibernate during the winter months, but the malarial plasmodium lives through to the next spring in the blood of malarial subjects; so it is virtually the man, and not the mosquito, that carries over this disease from one season to another.

"Dr. Sevol Harris says malaria, though decreasing in severity and frequency, continues as one of the most prevalent diseases in many localities in the United States. In 1900 the number of deaths reported from malaria was 14,900. The seriousness of the disease and the importance of medical cure should be more emphasized, especially as quinine, taken long enough, is almost a specific for the disease. The most marked effect of malaria is seen upon the renal organs and vascular structures; consequently, in malarial countries, the death rate is high and the mortality from nephritis and various forms of paralysis is inordinately increased. There would be little chronic malaria if acute cases were cured, but there are today probably two or three million persons in the United States who are harboring malarial parasites. The microscope is not always a criterion to chronic malaria; undoubtedly, the disease exists without the presence of the parasites in the peripheral circulation. There should be a campaign of education begun with the medical profession, because physicians as a class do not appreciate the seriousness of malaria or the ease with which it can be eliminated.

"It has been reduced 65 per cent in five years in Italy by the free use of quinine. All duty on quinine or Peruvian bark should be removed by all countries. Boards of health should furnish the drug to poor people free in malarial sections. If we had the power to cinchonize our infected population in the winter time, and to drain our swamps, lagoons, marshes, and oil our lakes, we could get rid of malaria altogether. But the most optimistic can hardly expect this consummation at least during our generation.

"Within a period of twenty years as a practitioner and teacher of medicine in this city, I have only personally witnessed one death from malarial fever, and that was of the congestive or estivo-autumnal type at Ruddock, La., a point forty miles above the city.

"The anophiles is a back-of-the-city, country, lake or swamp-bred mosquito, and is not very prevalent in New Orleans, except on the lake border or where there is stagnant water. I think that the blood of three-fourths of our inhabitants of this city would be negative as to the malarial plasmodium. We very rarely see malarial chills in this city, except from patients who have migrated from the country. So far as the effect of malaria on longevity in New Orleans is concerned, I would consider it of no very great importance. The parishes along the river front are less infested with malaria than are the lands on the lakes, bayous and lagoons that exist further back in the swamps. This has caused the majority of insurance companies to remove the five and ten dollar extra per thousand which they originally charged against applicants living in the swamp section of the South, and very many of these companies have gone into previously prohibited territory to solicit business. This is dependent upon two causes. A great many of these farms and sections of swamp lands have been better drained, houses and beds have been screened; even our colored population, who are not very subject to malaria, know the preventive effect of the free use of quinine. So the numerous deaths formerly recorded from congestive malarial fever and malarial hematuria have given place to the milder forms of chills and fever, which are only harmful to longevity from its destruction of red-blood corpuscles, producing anemia, congestions of the liver and spleen, etc., rendering the patient less vulnerable to other diseases, or coming in as complications to pneumonia, Bright's, and any of the acute infectious diseases."

---

In an injury to the wrist, tenderness just distad to the radius and in the "anatomical snuff box" is significant of fracture of the scaphoid.—*American Journal of Surgery.*

